# Telemedicine and Telementoring in Urology: A Glimpse of the Past and a Leap Into the Future

**DOI:** 10.3389/fsurg.2022.811749

**Published:** 2022-02-22

**Authors:** Christian Habib Ayoub, Jose M. El-Asmar, Suhaib Abdulfattah, Albert El-Hajj

**Affiliations:** ^1^Department of Surgery, Division of Urology, American University of Beirut Medical Center, Beirut, Lebanon; ^2^American University of Beirut Medical School, American University of Beirut, Beirut, Lebanon

**Keywords:** telemedicine, telementoring, urology, robotic, telerobotic, telecommunications, COVID-19, education

## Abstract

Telemedicine is the process of utilizing telecommunications and digital relay to perform, teach, or share medical knowledge. The digital era eased the incorporation of telemedicine to different areas of medical care, including the surgical care of Urologic patient mainly through telementoring, telesurgery, and telerobotics. Over the years, Telemedicine has played an integral part in a physicians' ability to provide high quality medical care to remote patients, as well as serve as an educational tool for trainee physicians, in the form of telementoring. During the COVID-19 pandemic, telemedicine has played a vital role in combatting the health implications of confinements. Challenges of telemedicine implementation include cost, ethical considerations, security, bandwidth, latency, legal, and licensure difficulties. Nevertheless, the future of telemedicine, specifically telementoring, promises several improvements and innovative advancements that aim to bridge the gap in technological divides of urologic care. In this review, we build on what is already known about telemedicine focusing specifically on aspects related to telementoring, telestration, and telesurgery. Furthermore, we discuss its historical role in healthcare with a special emphasis on current and future use in urology.

## Introduction

Telemedicine can be defined as any technology or technique that uses telecommunications or any form of remote interactions for medical interventions (1); it is the virtual conveyance of healthcare related information between two distinct sites. Telemedicine encompasses several sub-categories that include but are not limited to: Telecommunications, Telementoring and Telesurgery (2). Telementoring is further divided into Teleproctoring, Telestration and Teleassistance (2). Teleproctoring entails verbal guidance by a mentor to a trainee. Telestration enhances the interaction by allowing the mentor to indicate or draw on a live feed during an intervention or procedure, while Teleassistance permits the remote surgeon to have direct access to some of the instruments involved in the procedure (2). On the other hand, telesurgery is the independent conduction of surgery by a remote surgeon (3, 4). In the past years, the application of telemedicine has seen significant growth among healthcare workers. In 2020, the European telemedicine market was valued at $10.6 million, and it is expected to reach around $30 million by 2026^1^ (5). Approximately 15 million Americans receive remote medical assistance yearly (6). In this review, we build on what is already known about telemedicine focusing specifically on aspects related to telementoring, telestration, and telesurgery. The purpose of this review is to highlight its historical role in healthcare with a special emphasis on current and future use in urology. We further discuss the role of telementoring and telesurgery in everyday urologic care emphasizing their benefits and limitations.

## Methods

**Figure 1:**
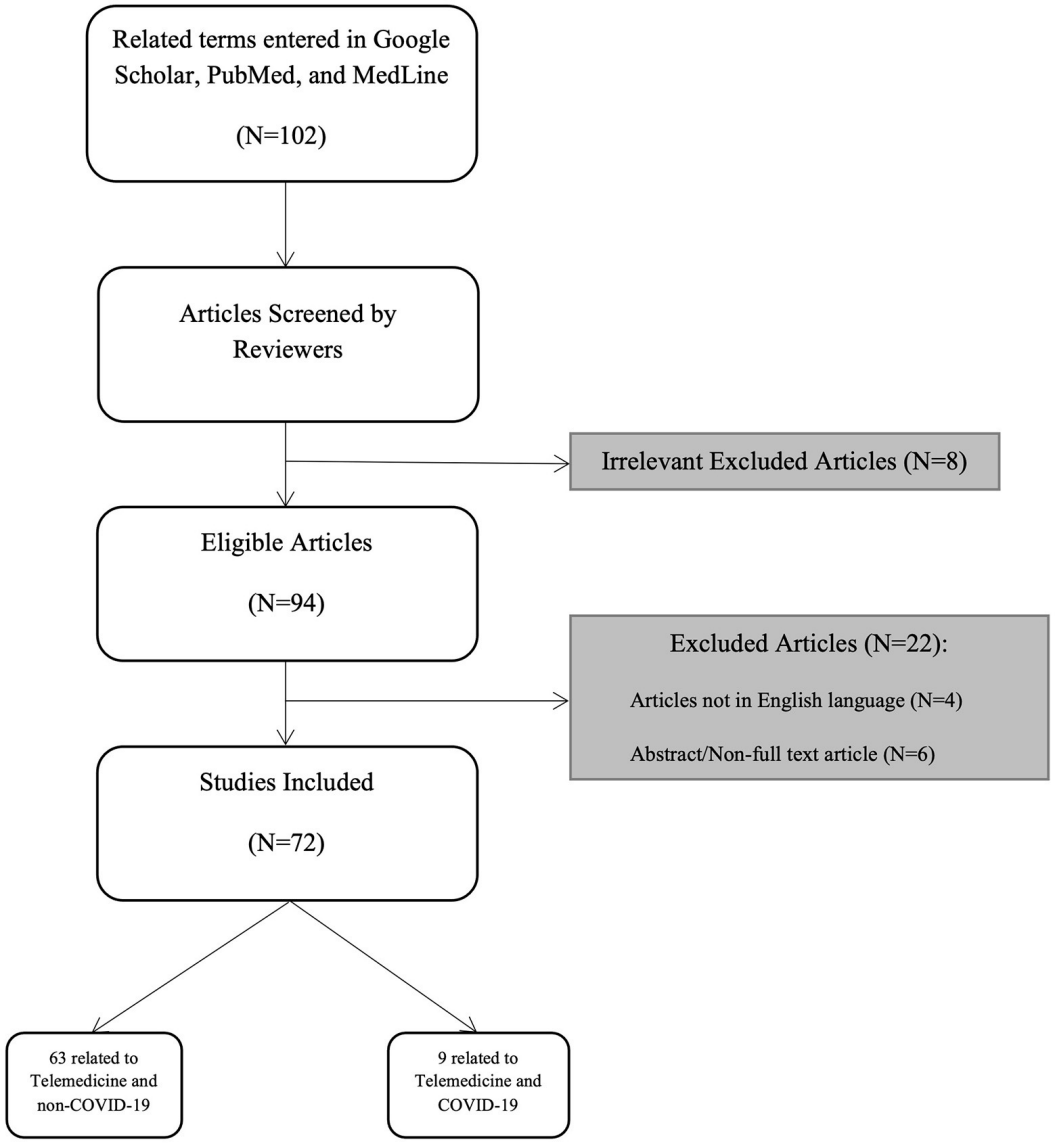
Flowchart illustrating the search strategy used in this review.

We performed an extensive search in Google Scholar, PubMed and MedLine using the terms “urology”, “urologic surgical procedures”, “urolog^*^ method^*^” combined with one of the following terms: “telemedicine”, “telemedic^*^”, “telecommunicat^*^”, “telestration”, “telerobot^*^”, “remote mentoring”, and “education”. For the section on COVID-19 we added to our previous search “COVID-19^*^, “COVID” and “Pandemic”. We inlcluded original articles, systematic reviews, and brief reviews written in the English langauge. We focused on addressing articles that discussed telementoring and telesurgery. Articles about telerounding, teleconsultations, and televisits were beyond the scope of this paper, hence were excluded. A total of 102 articles were screened of which 94 were found to be relevant and 74 were included in this review whereas 9 of those addressed the topic in relation to COVID-19 (Figure 1). We sough to write a brief narrative, non-systematic review discussing telementoring, telestration, and telesurgery in urology.

### History of Telemedicine

An early application of Telemedicine dates back to 1903 when Willem Einthoven, father of electrocardiography, successfully achieved a trans-telephonic transmission of an electrocardiograph to a distant hospital (7). Since 1929, laparoscopic techniques were introduced, and their use was first investigated in Urology and General surgery (8). The adoption of laparoscopic and robotic techniques in urologic surgeries eased the use of distant interactions between two remote sites. The invention of Automated Endoscopic System for Optimal Positioning (AESOP) in 1993 (9), Probot in 1996 (10, 11), Percutaneous Access to the Kidney (PACKY) in 1997 (12), Zeus, and later the DaVinci robots made such interactions possible (13). Those inventions paved the way for laying the foundations of telesurgical platforms.

One of the earliest uses of telementoring, specifically telestration, was done by Moore et al. (14) in 1995. An experienced surgeon mentored a trainee, 1,000 feet apart, to perform 23 laparoscopic procedures by utilizing high resolution video feeds, 2-way audio connections, telestration platform, and AESOP robot. 1 year later, a similar set-up was developed for telesurgical consultations and was successfully used to perform 6 complex laparoscopic surgeries (15). In 1996, the first attempt at teleassistance was done by Schulam et al. (16) who utilized a newer version of the AESOP (1,000 TS) robot granting the remote surgeon full control of the electrocautery devices during the procedure. All these attempts were successful and showed comparable results between on site and distant telementoring (14–16). In the following years, further improvements to the field of telementoring and telemedicine were introduced. Between 1998–2000, Bove et al. (1) successfully performed 17 transcontinental telementored and telestrated procedures between Italy and USA using AESOP and PAKY through Integrated Services Digital Network (ISDN) telephone lines rather than fiber optic lines. This aimed at simplifying connectivity issues between the sites. Nevertheless, in the initial pilot study, 5 out of 17 cases faced connection difficulties to the remote site, due to high bandwidth, halting the procedure. The authors concluded that a full adoption of telementoring procedures could only be established after significant advancements in the telecommunication systems (1).

The introduction of Zeus and DaVinci robots marked a breakthrough in the use of telesurgery. These robots received FDA approval in early 2,000 and were quickly used in general and urologic surgeries. The first robotic tele-cholecystectomy was successfully performed by Jacques Marescaux et al. (17) using the Zeus Robot and transatlantic optical fiber network between France and New York, known as “The Lindbergh Operation”. It was deemed as a successful, reliable procedure with no perceived lag (17). In urology, Sterbis et al. (18) used public internet connectivity to perform the first transcontinental use of a DaVinci robot to perform 4 right nephrectomies on Porcine models that were 1,300 and 2,400 miles away, latency times was minimal between 450 to 900 ms. These historical events paved the way for the integration of telemedicine in every day medical practice.

### Telementoring: Teleproctoring and Telestration

The ability to safely guide a trainee during the learning phase of a procedure has been at the core of surgical education. To do so remotely is the essence of Telementoring. Telementoring encompasses Teleproctoring and Telestration.

Teleproctoring requires a trainer and a trainee to be connected through an interface that allows direct communication between the two. The interface requires two essential components: a digital platform and a connection between the two sites.

*Connections* either depend on cable or wireless systems as means of information relay. Over the years, those systems have developed to enhance the learning experience. At first, an ISDN and dedicated trunk communication lines were used (1, 16), followed by the use of cable networks in the form of dedicated fiber optic channels that provided a faster bandwidth and a decreased lag time (17). Further improvements in the field of telecommunications led to the use of wireless networks such as local internet connectivity. They were considered a means for practical and inexpensive telemedical communications (18). Today, wireless internet networks have rapidly evolved into providing unprecedent levels of data transfer speeds leading to the 5th generation technology standard for broadband of cellular networks, known as 5G. Mobile cellular networks satellites have been efficiently used in various forms of telemedicine with minimal connectivity lag (3).

*Digital platforms* of communication for teleproctoring and telestration have been developed to facilitate effective two-way communications between a mentor and his trainee. Such interfaces include a telementoring platform developed by Intuitive Surgical Inc. (Sunnyvale, CA, USA), called Connect^TM^ and a telemetry system developed in Boston, London, and Beirut, called Proximie^TM^. The aim is to permit virtual scrubbing of mentors to guide trainee surgeons by assisting in all aspects of the procedure including incision sites, anatomical identification etc.

Shin et al. (19) used Connect to mentor 55 robotic prostate and renal surgeries of which 29 were onsite and 26 were telementored. The interface provided effective telestration on the operative screen using the robotic console over a hospital-based internet connectivity. The results of this study showed that telementoring was simple and effective allowing for minimal blood loss with similar operative times between telementored and non-telementored cases. Furthermore, higher satisfaction rates were reported by surgeons and trainees where mentors preferred remote over in-person interaction (19).

Similarly, a more recent study using another telementoring interface called Proximie compared perioperative outcomes of 59 cases of Aquablation, a novel robotic procedure to treat Benign Prostatic Obstruction, of which 39 were on site while 21 were telementored. This platform allows augmented reality live videos stream, low bandwidth internet connectivity, 2-way audio-video relay with zoom and encrypted web servers. It also allows the surgeon to draw on the displayed image to highlight important structures and anatomic landmarks. Similar operative outcomes and complications were seen between telementored cases and on-site cases demonstrating the practicality of such interfaces in teleproctored cases (20).

With innovative digital platforms and connections comes the ability to illustrate surgical steps on the surgical screen. *Telestration* adds a new dimension to telementoring whereby a trainer can telestrate his operative thoughts onto the field by identifying, delineating, or drawing on the displayed image to highlight important structures and anatomic landmarks during a procedure. This direct two-way interaction offered by Telestration has been shown to significantly improve the teleproctoring experience (4, 19).

### Telesurgery and Telerobotics

Telesurgery is defined as the ability to independently perform a surgical procedure remotely (9). On the other hand, tele-robotics is a subtype of telesurgery whereby robotic instruments are used to perform the surgery. In 1995, the field of Urology witnessed the first application of a full telesurgical approach when Rovetta et al. (26) performed the first telesurgical transrectal ultrasound-guided biopsy of the prostate. Initially, laparoscopic equipment were utilized in telesurgical procedures using the AESOP robot. The remote surgeon was only limited to camera control and some laparoscopic or electrocautery functions (1, 16). With the introduction of the Da Vinci robot, the remote surgeon was able to manipulate more equipment and in some cases perform a full telerobotic surgery (18). Telerobotic surgeries in Urology were initially introduced using the Da Vinci surgical system to perform remote nephrectomies on distant porcine models (18). While in fact one of the first examples of telerobotic surgery took place in the year 2002 between Baltimore and Munich during a laparoscopic renal cyst ablation (22). In 2020, the Da Vinci surgical system was used to perform 1.25 million procedures, a 1% growth compared to 2019 (23). It is expected that the robotic market in urology will witness a growth of 11.7% by 2027^2^ (24). Today, further improvements in surgical robots allowed the introduction of competitive systems to the market such as Senhace, Revo-I, Versius, Avater, Hinotori, and most recently the Hugo RAS (25). Data on these systems are still not well-established and human trials are ongoing. Nevertheless, promising advances in the telerobotics sector are expected.

## Discussion

### Benefits to Physician and Patient

Teleproctoring and Telestration have various advantages related to physician availability, omission of travel costs, combatting travel related restrictions, and emergency interventions specially in conflict areas with underdeveloped healthcare systems (Table 1). Rogers et al. (21) showed that telementoring between a community-based hospital and a trauma center resulted in 7% lifesaving consultations and more than 80% approval rating for improved patient care. In regards to the immediate aid of areas of conflict, telementoring was the sole solution for performing a complex hand reconstruction surgery for a blast injury patient in Gaza (4). It is estimated that five billion people do not have access to safe and affordable healthcare and that only 6% of the world's annual surgical procedures occur in the poorest countries (6). Using telementoring and telesurgery, patients in remote areas and underdeveloped countries can benefit from high quality healthcare. Physicians have shown various levels of satisfaction using telemedicine. In one study, mentors significantly favored remote telestration over in-room telestration (19). Furthermore, a survey study addressing the use of telementoring as a form of communication between residents and faculty physicians, revealed that most respondents expressed a moderate and strong agreement regarding the positive impact of telementoring in improving their interaction for the benefit of patients (27).

**Table 1:** Benefits of telementoring and telesurgery.

**Increases physician availability especially in emergencies**	**Improves resident training by allowing state-of-the-art surgical techniques and procedures to be taught remotely**
**Decreases travel hurdles and costs**	**Decreases the impact of pandemics and worldwide health crisis on delivering healthcare**
**Facilitates travel related complications and restrictions**	**Decreases educational gaps by allowing conferences, lectures and rounds to occur remotely**
**Allows widespread dissemination of high-quality healthcare especially in areas with underdeveloped healthcare**	**Increases interactions between physician and trainee by allowing physician to clearly telestrate crucial surgical steps in the trainee's surgical view**

### Telemedicine in Coronavirus 2019 Pandemic

In 2019, SARS-CoV-2, known as COVID-19, emerged in the city of Wuhan, China, spreading across all continents before being declared as a worldwide pandemic on 11 March 2020. This caused a major turning point by limiting human interactions affecting both their physical and mental well-being (28). COVID-19 presented various challenges to the public, administrative and healthcare sector (29).

COVID-19 left a drastic impact on the healthcare sector affecting the worldwide health care economy, as well as the physical and mental well-being of healthcare professionals (29). In a scoping review of the impact of COVID-19 on education and training, junior medical staff reported that the pandemic significantly limited their educational activities and training quests (30). This was mainly due to the sharp reduction of non-COVID-19 related patient encounters. (31). Urologist's interest in telemedicine increased during the pandemic from 43.7 to 80.8% with 81% interested to continue using telemedicine in their practice (32). Telemedicine was adopted in the pandemic as an easy and convenient alternative to mentor procedures without the need for travel. Furthermore, the American Urological Association urged urologists to optimize the use of telemedicine as it ensured the safety and well-being of their patients and workforce (33). An example of institutional use of telemedicine during the pandemic was the adoption of remote triaging by symptom screening (cough, sore throat, fever, cold etc.) and epidemiological history taking (contact with a positive case) over the phone prior to patient presentation to the hospital (34). In addition, there was a heavy reliance on virtual softwares such as Microsoft Teams, Zoom, Cisco, Webex and Skype that were extensively used to bridge the gap between physicians and patients. Bokolo et al. (35, 36) suggested a step-by-step workflow model that orchestrates remote consultation. This enabled patients to receive medical attention and schedule follow-up appointments. In fact, authors predicted their utility to last long after the end of the pandemic (35). In addition, efforts were made to lay the foundations for a remote interaction that is safe and governed by a set of practice guidelines and recommendations (36). Other studies demonstrated the successful use of telemedicine in selected patients during COVID-19 in the diagnosis, management, and follow-up care of oncologic and non-oncologic urologic diseases (37).

### Telemedicine and Education/Training

In the wake of the COVID-19 pandemic, surgical residents reported a significant decline in their educational and training exposure (30, 38). Such decline was due to a decreased number of patient case load, as well as the cancellation of elective surgeries (39). In addition, educational activities such as lectures, grand rounds, and conferences were halted. Telemedicine aimed to combat those repercussions through offering an alternative means of telecommunication. Furthermore, physicians travel long distances to attend conferences, symposiums, or hands-on training courses to be up to date with the latest guidelines and novelties in the field. This leads to an increase in travel and logistical expenses. Telemedicine offers a way to ease knowledge gaps and make surgical training accessible without the need for travel (40).

As was previously stated, telemedicine can facilitate training collaborations between remote institutions allowing experienced surgeons to guide junior doctors in performing complex surgeries (41). For instance, one study demonstrated that telementored residents performed as well as non-telementored residents during surgical training (42). Annual caseload affects surgical proficiency whereby an increase in case load increases proficiency (43). Telemedicine can fill the case load shortage through allowing urology trainees to virtually scrub in on cases to enhance their case load exposure. In addition, surgeons seeking mastery of minimally invasive surgeries can immensely benefit from telemedicine. For instance, the learning curve of a radical prostatectomy is around 250 cases, while more than 80% of surgeons perform less than 10 cases a year (44). As such, the ability to safely master this procedure can make use of a guiding hand that is able to remotely assist when needed. Telemedicine can act as the bridging gap for the occasional need of an expert opinion to surpass a challenging surgical step.

### Challenges of Telemedicine

**Figure 2:**
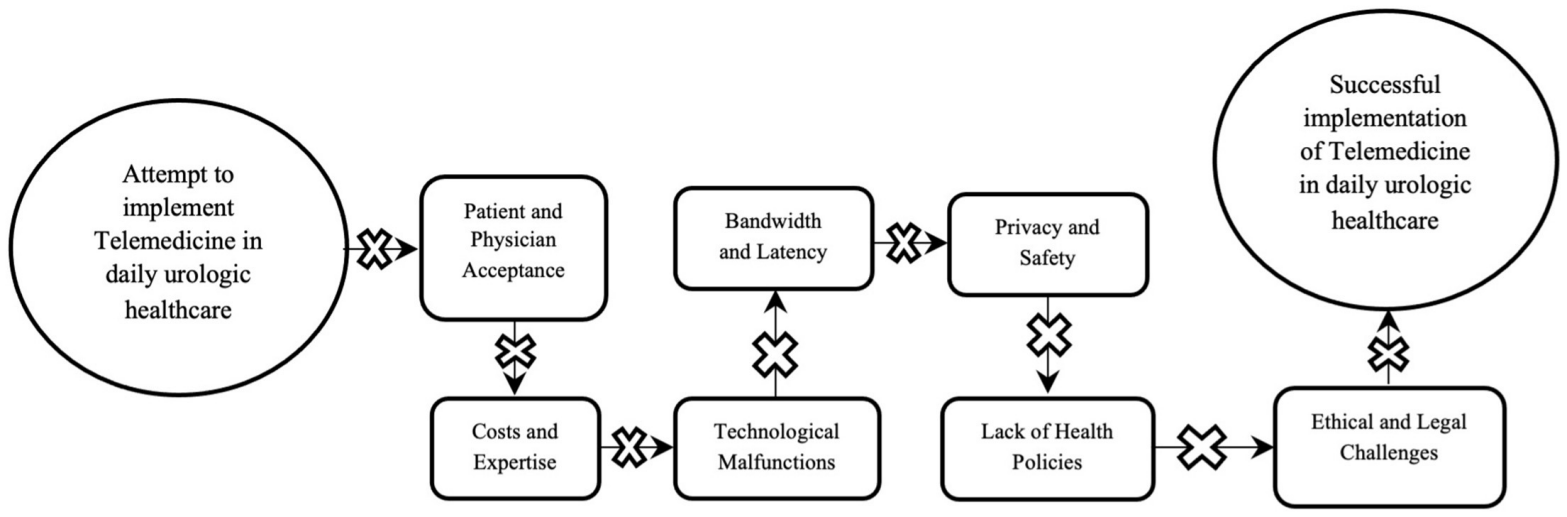
Illustration depicting the obstacles of implementing telemedicine in urology.

Telemedicine was found to be useful and effective in healthcare settings; nevertheless, it presents with limitations and challenges (Figure 2). Telemedicine is yet to be widely accepted in the urological community due to several limitations pertaining to patient and physician acceptance, licensure and liability, costs, safety, ethical considerations, and changes in workflow (45, 46).

In some cases, patients reported differences in quality of care and hesitancy in using such novel techniques in healthcare (45). On the other hand, healthcare providers reported lack of experience in using these technologies, resistance toward detachment from physical patient interactions, and inconvenient changes in staff and workflow (45). In addition, physicians faced licensure dilemmas and financial obstacles due to telemedical use across borders leading to an inadequate distribution of liability between mentor and trainee (47).

Telemedical practices incur heavy costs. As an example, robotic devices require an investment and running cost of around 800,000$ and another 100,000$ per year in maintenance fees. Not many hospitals can afford such expenses (48). On the other hand, on-site-mentoring incurs travel costs, patient lost time, and physical presence of the physician, whereas telementoring allows these expenses to be offset at the expense of buying tools for telecommunications (49). Further studies are required to determine the cost-benefit relationship of telemedicine.

Technology presents with various pitfalls. Machines are vulnerable to malfunction and in certain situations, such malfunctions could be catastrophic. Bove et al. performed 17 telementored procedures using AESOP and PAKY between Rome and the USA. In these 17 procedures, five were dropped from telementoring due to connection difficulties, two converted to open due to intra operative complications, and two faced intraoperative technical robotic malfunctions (1). Furthermore, various telementored and telesurgical attempts reported equipment malfunction and internet instability causing complications in their attempts (18, 50, 51). Bandwidth and latency hurdles are also limitations of telesurgical and telementored procedures. The amount of delay, connection stability, and integrity of the surgical/telementored experience relies on an adequate bandwidth with minimal latency. To provide minimal latency times, bandwidth, known as the rate of data transfer between two points, should be sufficient. Previously, bandwidth speeds depended on the type of data connection, distance of data transfer and overall traffic in the circuit. Nowadays, due to 5G, bandwidth speeds have reached 1Gb/s allowing short latency times and almost instant surgical and telementored relays (3). The exact amount of latency permitted is yet to be established, but various studies suggested that in order to maintain surgical performance latency times should not exceed a range of 330–450 ms (18, 41, 52). A final drawback to telemedicine is privacy and safety. Hacking and cyberattacks are concerns to tele-robotic networks, leading to concerns for the safety and vulnerability of data transfer (53). Breaches to these networks allow access to confidential patient data and privacy. Faster wireless connections, reliable firewalls, and virtual private networks (VPNs) are amongst the safety implementations needed to resolve these concerns.

### Telemedicine and Health Policy

Novel approaches to healthcare, such as telemedical practices should be defined and regulated by regional and international policies. Health policies and telemedicine are interconnected and for telemedicine to be effective, regulated and fair between patients, health policies should be drafted and applied. Telemedicine offers innumerable healthcare advantages including easy access, convenience, self-efficacy, cost-cutting, and improvement in the overall population health (54). These advantages should encourage policy makers to enforce regulations that minimize the obstacles of telemedicine. Licensure between different states is one of the main obstacles faced (54). In the United States, the Federation of State Medical Licensing Board has erected the Interstate Medical Licensing Compacture that aimed to combat interstate variability by ensuring expedited licensure for the remote physician (55, 56). In addition, state specific policies were also drafted by the American Telemedicine Resource Center in an attempt to define and unify telemedical cross state interactions^3^ (57). Nevertheless, there exists a lack of unified and robust national and international policies allowing providers to provide healthcare across borders (54). Other obstacles to be tackled by health policy makers are reimbursement controversies and patient information privacy (54). Policies such as the Affordable Act Care (ACA) and Health Information Technology for Economic and Clinical Health (HITECH) Act have attempted to address such limitations by ensuring fair reimbursements and securing that patient information remains safe and confidential (58, 59). It is without doubt that health policies regulating telemedicine improved dramatically over the past couple of years, yet further improvements are still due.

### Legal Challenges of Telementoring and Telesurgery

Telemedical practices such as telementoring and telesurgery are more effective when allowed to supersede limitations imposed by geographical boundaries. The fact that telemedical encounters often occur across borders lead to several legal dilemmas. In the United States, state specific physician licensing is a major legal hurdle against the adoption of telemedicine across state borders; hence, the ability to treat patients or telementor procedures require different licenses in different states (45). As an illustration, several states mandate that remote physicians need to hold state specific licenses prior to treating patients in those states (60). Furthermore, some states restrict telemedical encounters to follow-up visits only, hence patients must first attend to an in-person visit before telemedical encounters are allowed (46). It is worth noting that health policies and licensing organizations have allowed expedited licensure for remote physicians (55, 56). In fact, state specific policies and regulations for physicians treating patients across state borders can be found in the American Telemedicine Association Resource Center^3^ (57). However, there still lacks a unified legal reference to define multistate licensing and to organize cross-border physician practices (49, 61). On the other hand, telementoring and telesurgery pose serious ethical considerations in regards to the proper attainment of a patient's informed consent. Consent for the telementored or teleoperated procedure should be clear, specific, and detailed informing the patient of the risks and benefits of such a procedure (62, 63). The procedure or intervention should be clearly described making sure that the explanation is clear, understandable, unambiguous and explicitly addresses the shortcomings of a tele approach (63, 64). Further legal obstacles that can complicate a telemedical procedure includes data protection, physician malpractice, and physician liability (61). Some authors believe that health informatics professionals (HIPs) who are responsible for the management, security, and implementation of healthcare informatics systems should be accountable by laws and codes in regards to data protection and breaches related to telemedical practices (65). A report by the Identity Theft Resource Center (ITRC) showed that the medical/healthcare sector contributed to 34.5% of the total amount of data breach incidents (66), thus data transfer over virtual networks during telementoring and telesurgery render the process significantly hackable. Several legislations and laws were drafted to protect patient data and security such as the General Data Protection Regulation, Directive 2011/24/EU, and others (67). Nevertheless, gaps regarding medical liability, security, and legal concerns need to be addressed and standardized prior to universal adoption of telemedicine (67).

## Future Directions

The digitalization of todays' world enables a future global integration of telemedicine. Telemedicine and Telementoring have a prosperous future based on the initial studies that proved their efficiency and ease of implementation. Subsequently, telementoring using augmented reality (AR) and wearable equipment will be possible. Such attempts have already been materialized using holographic glasses such as Google Glass and Microsoft HoloLense. These models were found to be effective in remote telementoring yet require human trials to further assess their efficiency (68, 69). The telesurgical and telerobotic industry witnessed the introduction of various novel robotic systems to the market. These new robots feature significant improvements and advances in their setup. Some improvements include single port entries, smaller patient carts, open surgeon consoles with augmented reality, haptic feedback, and separated robotic arms (25). These new robots have been found to be effective in many surgeries and have provided benefits as compared to more traditional machines (70–72). Nevertheless, further trials and studies are required to prove the advantages of such changes to telerobotics.

## Limitations

This review has several limitations. To start, we limited our search to telementoring, telesurgery, and telerobotics. Hence, we have left out other uses of telemedicine in urology such as teleconsultations, telehealth, and telerounding. The uses of telehealth were only mentioned when discussing the role of telemedicine during the COVID-19 pandemic. The aim was to focus on telementoring and telesurgery, two important pillars in remote medical care; hence, refraining from further exploring into the uses and implications of the other subtypes of telemedical care. The following manuscript is a brief narrative, non-systematic review of what is already known regarding telemedicine in urology. Broader categories of telemedicine and related publications were beyond the scope of this paper. In addition, we searched three major databases: PUBMED, MEDLINE and Google Scholar leaving out other databases that might have contributed to our paper.

## Conclusion

Telemedicine has the potential to break geographical limitations of medical care. It is an exciting and promising field with boundless opportunities. The implementation of telemedical practices can greatly impact the quality of patient care, in addition it serves as a tool for enhancing the training of resident physicians and combatting pandemic related confinements. There are still several barriers impeding a full integration of telemedicine such as cost, ethical considerations, security, bandwidth, latency, and licensure difficulties. Nevertheless, the future of telemedicine, specifically telementoring and telesurgery, promise several improvements to tackle those barriers.

## Author Contributions

CA conceptualized the study, retrieved, read, and summarized the articles, and wrote the manuscript. JE–A conceptualized the study, retrieved and summarized the articles, and wrote the manuscript. SA retrieved and summarized the articles and wrote the manuscript. AE–H reviewed and edited the manuscript and supervised the review and writing process. All authors contributed to the article and approved the submitted version.

## Conflict of Interest

The authors declare that the research was conducted in the absence of any commercial or financial relationships that could be construed as a potential conflict of interest.

## Publisher's Note

All claims expressed in this article are solely those of the authors and do not necessarily represent those of their affiliated organizations, or those of the publisher, the editors and the reviewers. Any product that may be evaluated in this article, or claim that may be made by its manufacturer, is not guaranteed or endorsed by the publisher.

## Footnotes

^1^ https://www.mordorintelligence.com/industry-reports/global-telemedicine-market-industry

^2^ https://www.databridgemarketresearch.com/reports/global-urology-robotic-surgery-market

^3^ https://www.americantelemed.org/

## Abbreviations

COVID-19: Coronavirus Disease of 2019
AESOP: Automated Endoscopic System for Optimal Positioning
PAKY-RCM: Percutaneous Access to the Kidney Remote Center of Motion
ISDN: Integrated Services Digital Network
AUA: American Urology Association
ACA: Affordable Act Care
HITECH: Health Information Technology for Economic and Clinical Health
HIP: Health Informatics Professional
ITRC: Identity Theft Resource Center.
